# Outcomes of prior cervical cytology and HR-HPV testing in women subsequently diagnosed with CIN1, CIN2/3, and invasive cervical cancer: a 4-year routine clinical experience after implementation of systematic training and quality control programs

**DOI:** 10.1186/s12885-020-07321-2

**Published:** 2020-08-26

**Authors:** Dongman Zhao, Liran Zhang, Fengxiang Xie, Dezhi Peng, Jie Wei, Lingbo Jiang, Shoudu Zhang, Debo Qi

**Affiliations:** 1 Department of Pathology, Jinan KingMed Diagnostics, Jinan, 250101 Shandong Province China; 2 Department of Laboratory Medicine, Jinan KingMed Diagnostics, Jinan, 250101 Shandong Province China

**Keywords:** Cervical intraepithelial neoplasia, Cervical cancer, Screening, Cervical cytology, High-risk human papillomavirus (HR-HPV), Co-testing, Quality control, Training

## Abstract

**Background:**

In 2013, Jinan KingMed Diagnostics (JKD) first established a systematic cervical cytology training and quality control (QC) program in Shandong Province, China. We compared the efficacy of high-risk human papillomavirus (HR-HPV) detection, cytology, and their combination in routine clinical practice after the implementation of the training and QC program to identify the optimal first-line screening method in this region.

**Methods:**

The data of patients histologically diagnosed with cervical intraepithelial neoplasia (CIN) 1, CIN2/3, and invasive cervical cancer (ICC) between January 2014 and December 2017 were retrieved from the JKD database. Cytology and/or HR-HPV testing results within 3 months preceding the CIN1 diagnoses and 6 months preceding the CIN2/3 and ICC diagnoses were analyzed.

**Results:**

Prior screening data were available for 1829 CIN1 patients, 2309 CIN2/3 patients, and 680 ICC patients. Cytology alone and HR-HPV testing alone had similar rates of positive results for CIN2/3 (97.2% [854/879] vs. 95.4% [864/906], *P* = 0.105) and ICC detection (89.1% [205/230] vs. 92.7% [204/220], *P* = 0.185). Compared with either method alone, co-testing slightly increased the screening sensitivity for CIN2/3 (99.8% [523/524], all *P* < 0.001) and ICC (99.6% [229/230], all *P* < 0.001) detection. In the CIN1 group, cervical cytology alone (92.9% [520/560]) was more sensitive than HR-HPV testing alone (79.9% [570/713], *P* < 0.001), and co-testing (95.3% [530/556]) did not significantly improve the screening sensitivity (*P* = 0.105).

**Conclusions:**

After the implementation of a systematic training and QC program, both cytology and HR-HPV testing may be adopted for primary cervical cancer screening in Shandong Province.

## Background

Cervical cancer is the third most common malignancy in women, and approximately 85% of all cases of cervical cancer occur in low-resource countries, where there is a lack of trained personnel for cervical cancer screening [1]. Cervical cancer mainly develops from precancerous lesions, namely, cervical intraepithelial neoplasia (CIN) [2, 3]. The transformation from CIN to invasive cancer generally takes about 5 to 10 years [2, 3]. If these precancerous lesions are obliterated, the occurrence of most cases of invasive cervical cancer (ICC) can be effectively prevented. Over the past several decades, conventional Papanicolaou smear (CPS) has been used as an efficient, cost-effective screening method for the prevention and early diagnosis of cervical cancer. In the United States and most other developed European countries, systematic training programs for cytotechnologists and/or cytopathologists as well as detailed regulations governing cytopathological quality control (QC) processes are well-established to ensure the efficacy of screening [4, 5]. After the implementation of these training and QC provisions, the incidence and mortality of cervical cancer has drastically reduced in the last 60 years [6–8]. At the beginning of this century, the cytological efficiency of cervical cancer screening was further improved with the widespread application of liquid-based cytology (LBC) [9, 10].

Persistent high-risk human papillomavirus (HR-HPV) infection, especially with the HPV-16/18 genotypes, is the leading cause of cervical cancer and its precancerous lesions [2, 11]. Initially, HR-HPV detection was used for the further triage of abnormal cervical cytological results that could not be clearly interpreted [12]. However, clinical studies [13, 14] have shown that adding HR-HPV testing to cytology can increase the detection rate of cervical cancer and its precursor lesions in the screened populations. Based on these findings, HR-HPV and cytology co-testing was recommended as the primary screening modality for women aged 30 to 65 years [15]. In recent years, however, several large, randomized controlled clinical studies have revealed that HR-HPV testing alone can detect more cases of cervical cancer and its precancerous lesions than cytological screening [16–19]. The ATHENA study has further confirmed that the detection rate of CIN3 and above lesions by HR-HPV testing with separate HPV16 and HPV18 detection was comparable to that of a single cytological screening test [20]. Based on the above results, HR-HPV testing was approved as a first-line method of cervical cancer screening in several developed countries [21, 22].

Cervical cancer is highly prevalent in China. In 2012, Chinese women accounted for 12% of all new cases and 11% of all deaths due to cervical cancer in the world [23]. Both the incidence and mortality rates of cervical cancer in China have been showing year-on-year increases since a decade [23, 24]. Furthermore, due to the lack of a standardized cancer registration system, the cervical cancer incidence and mortality rates in China, especially in suburban and rural areas, might have been underestimated. Although some large-scale cervical cancer screening programs have been carried out in China, there is a lack of qualified cytopathologists, and cervical cancer screening is often not viable in rural or low-resource areas [25, 26]. Currently, China has not yet established a well-organized training system and unified cervical cytology QC standards for cervical cancer screening [25, 26]. These factors might have resulted in low screening efficiency of cervical cytology. In recent years, HR-HPV detection alone as well as HR-HPV and cytology co-testing were applied for routine cervical cancer screening in the as yet largely unscreened Chinese population. Presently, three modalities are available for cervical cancer screening: cytology alone, HR-HPV testing alone, and HR-HPV and cytology co-testing. However, no consensus has been reached about which of these is the optimal first-line screening method for cervical cancer prevention.

KingMed Diagnostics (KD) is the largest independent operator of pathology laboratories in China, and has established China’s first ever training school for cytopathologists and formulated cytological QC measures at its headquarters in Guangzhou, in accordance with the College of American Pathologists (CAP) requirements [10]. Jinan KingMed Diagnostics (JKD), a local pathology laboratory of KD in Shandong Province, has conducted a similar cytopathologist training and QC program since 2013. After the implementation of these measures, the abnormal cervical cytology reporting rates significantly increased in large-scale test programs involving the CPS or LBC method, suggesting that cytopathologist training and QC programs significantly improve screening efficacy [9]. In this study, we further compared the screening efficiency of LBC testing alone, HR-HPV detection alone, and their combination in patients who subsequently received histological diagnoses of CIN1, CIN2/3, and ICC. All screening tests were performed over a 4-year period after the implementation of a systematic training and QC program. The aim of this study is to provide further scientific basis for the establishment of a systematic training and QC program, and to guide the selection of the optimal primary screening method for cervical cancer prevention in an underserved population from Shandong Province, China.

## Methods

### Patient cohort

JKD provides clinicopathological services to more than 900 hospitals, physical examination centers, and community clinics throughout Shandong Province. After a formal approval by KD’s ethics review board, patients with histological diagnoses of CIN1, CIN2/3, and ICC were identified from the pathology databases of JKD over a 4-year period from January 2014 to December 2017. In this study, we included only the cytology and/or HR-HPV testing results obtained within the 3 months preceding a CIN1 diagnosis or within the 6 months preceding a CIN2/3 or ICC diagnosis. The majority of the cases included in this study were collected from local hospitals, physical examination centers, and community clinics that serve mainly suburban and rural areas, where a large number of clinicians are not specially trained or qualified. Cytology alone, HR-HPV testing alone, and co-testing with cytology and HR-HPV testing were all used as screening modalities. The diagnoses of CIN1, CIN2/3, and ICC were rendered by histopathological examinations, including cervical biopsy, endocervical curettage, diagnostic excisional procedures, and hysterectomy. All LBC and histopathological examinations were performed at the Pathology Department of JKD, while HR-HPV detection was performed at the Molecular Department of JKD. Cytology and/or HR-HPV testing data from other hospitals or laboratories were not included in this study.

### Cytology preparation and interpretation

LBC preparation was carried out strictly in accordance with the manufacturers’ instructions [9, 27, 28]. All cytological examinations were reported using the terminology of the 2001 Bethesda System. The cytological interpretations were divided as follows: unsatisfactory specimen, negative for intraepithelial lesion or malignancy (NILM), atypical cells of undetermined significance (ASC-US), low-grade squamous intraepithelial lesion (LSIL), atypical squamous cells-cannot exclude high-grade squamous intraepithelial lesion (ASC-H), high-grade squamous intraepithelial lesion (HSIL), atypical glandular cells (AGC), and cervical cancer cells. We conducted a rigorous and systematic training and QC program for cytology processes, as we have previously reported [9, 10].

### HR-HPV testing

JKD has established the largest standardized molecular laboratory in Shandong Province and has obtained the International Organization for Standardization certification for molecular diagnosis to ensure the diagnostic accuracy of molecular testing. Nearly 200,000 samples are tested for HPV every year in the JKD laboratory. In this study, all HR-HPV tests were carried out in the standardized molecular laboratory of JKD by using one of two methods: Hybrid Capture 2 (HC2; Qiagen, Hinden, Germany) and HPV genotyping (Yanengbio, Shenzhen, China) [27–29]. The HC2 assay is an in vitro nucleic acid hybridization method, which can semi-quantitatively test for 13 HR-HPV genotypes (i.e., 16, 18, 31, 33, 35, 39, 45, 51, 52, 56, 58, 59, and 68). The HPV genotyping assay is an in vitro diagnostic kit using PCR-reverse dot blot hybridization measurement, and it can detect 14 HR-HPV genotypes (i.e., 16, 18, 31, 33, 35, 39, 45, 51, 52, 56, 58, 59, 66, and 68) and 9 low- or uncertain-risk HPV genotypes (6, 11, 42, 43, 53, 73, 81, 82, and 83). Only infections with one or more of the 14 HR-HPV genotypes were considered as a positive HR-HPV test result in this study.

### Statistical analysis

Statistical analysis was conducted using SPSS software (version 19.0, IBM Co., Chicago, Illinois, USA). The Pearson χ^2^ test was used to compare differences in categorical data, and the one-way analysis of variance were used to compare differences in continuous data, with the Bonferroni test being carried out where appropriate. *P* < 0.05 was considered statistically significant.

## Results

### Patient characteristics

During the 4-year study period, CIN1, CIN2/3, and ICC were histologically diagnosed in a total of 1829 patients, 2309 patients, and 680 patients, respectively, who had undergone prior cytology and/or HR-HPV testing (Table 1). Of the 680 patients with ICC, 585 (86.0%) patients had squamous cell carcinoma, 86 (12.6%) patients had adenocarcinoma, and 9 (1.3%) patients had adenosquamous carcinoma. The average age of the ICC patients was significantly higher than those of the CIN1 (*P* < 0.001) and CIN2/3 patients (*P* < 0.001), but no significant difference was observed between the average ages of the CIN1 and CIN2/3 patients (*P* = 0.846). In each of these three groups, no significant differences in average age were found between the different screening modalities.

**Table 1:** Ages of patients who underwent cytology alone, HR-HPV testing alone, and co-testing prior to the diagnoses of CIN1, CIN2/3, and ICC

Screening modality	CIN1		CIN2/3		ICC	
	No. (%)	Age, average (range), y	No. (%)	Age, average (range), y	No. (%)	Age, average (range), y
Cytology alone	560 (30.6)	41.0 (18–64)	879 (38.1)	42.5 (16–71)	230 (33.8)	49.0 (27–85)
HR-HPV testing alone	713 (39.0)	41.5 (14–66)	906 (39.2)	41.1 (20–67)	220 (32.4)	47.9 (25–88)
Cytology and HR-HPV co-testing	556 (30.4)	40.4 (17–70)	524 (22.7)	39.8 (18–72)	230 (33.8)	47.9 (28–87)
Total	1829 (100)	41.0 (17–70)	2309 (100)	41.3 (16–72)	680 (100)	48.3 (25–88)

*CIN* cervical intraepithelial neoplasia, *ICC* invasive cervical cancer, *HR-HPV* high-risk human papillomavirus

### Results of prior cytology alone

The detailed results of cytology alone are summarized in Table 2. The average interval between histological diagnosis and cytological testing was 21.8 days (range, 0–90 days), 20.5 days (range, 0–166 days), and 12.6 days (range, 0–160 days) in the CIN1, CIN2/3, and ICC groups, respectively. Overall, the rate of abnormal cytological findings was significantly higher in the CIN2/3 group (97.2%) than in the CIN1 (92.9%, *P* < 0.001) and ICC groups (89.1%, *P* < 0.001). However, this rate did not differ between the CIN1 and ICC groups (*P* = 0.083). LSIL was the most common abnormal cytological result in the CIN1 group (reported in 65.4% of patients), followed by ASC-US (20.5%), HSIL (5.2%), and ASC-H (1.8%). In contrast, HSIL was the most common abnormal cytological result in the CIN2/3 (52.7%) and ICC (48.3%) groups. The other abnormal results in the CIN2/3 group were LSIL (24.1%), ASC-H (12.2%), ASC-US (8.0%), cancer cells (0.2%), and unsatisfactory cellularity (0.1%). In the ICC group, cancer cells (16.1%) was the second most common cytological interpretation, followed by ASC-H (16.1%), NILM (8.3%), AGC (7.0%), ASC-US (5.2%), unsatisfactory cellularity (2.6%), and LSIL (0.4%).

**Table 2:** Results of prior cytology alone

Cytological interpretation	CIN1		CIN2/3		ICC	
	No.	%	No.	%	No.	%
NILM	40	7.1	24	2.7	19	8.3
Unsatisfactory	0	0.0	1	0.1	6	2.6
ASC-US	115	20.5	70	8.0	12	5.2
ASC-H	10	1.8	107	12.2	28	12.2
LSIL	366	65.4	212	24.1	1	0.4
HSIL	29	5.2	463	52.7	111	48.3
AGC	0	0.0	0	0.0	16	7.0
Cancer cells	0	0.0	2	0.2	37	16.1
Total	560	100.0	879	100.0	230	100.0

*CIN* cervical intraepithelial neoplasia, *ICC* invasive cervical cancer, *NILM* negative for intraepithelial lesion or malignancy, *ASC-US* atypical squamous cells of undetermined significance, *ASC-H* atypical squamous cells-cannot exclude HSIL, *LSIL* low-grade squamous intraepithelial lesion, *HSIL* high-grade squamous intraepithelial lesion, *AGC* atypical glandular cells

### Results of prior HR-HPV testing alone

Among patients who underwent only HR-HPV testing using the HC2 assay, the final histological diagnoses were as follows: CIN1, 309 (53.8%) patients; CIN2/3, 499 (55.1%) patients; and ICC, 69 (31.4%) patients (Table 3). The remaining patients who underwent HR-HPV testing alone underwent genotyping tests. The average interval between histological diagnosis and HR-HPV testing was 9.8 days (range, 0–90 days), 9.3 days (range, 0–146 days), and 6.5 days (range, 0–157 days) in the CIN1, CIN2/3, and ICC groups, respectively. The overall HR-HPV prevalence in the CIN2/3 (95.4%) and ICC (92.7%) groups was similar to each other (*P* = 0.112) but significantly higher than that in the CIN1 group (79.9%, all *P* < 0.001). The rate of positive HR-HPV results was similar for the two HPV testing methods in the CIN2/3 (HC2: 95.4% vs. genotyping: 95.3%; *P* = 0.996) and ICC groups (HC2: 94.2% vs. genotyping: 92.1%; *P* = 0.569). In the CIN1 group, however, HC2 testing resulted in a significantly higher rate of positive results than did genotyping (85.1% vs. 76.0%; *P* = 0.003).

**Table 3:** Results of prior HR-HPV testing alone

HPV test method	CIN1 (***n*** = 713)		CIN2/3 (***n*** = 906)		ICC (***n*** = 220)	
	Negative (%)	Positive (%)	Negative (%)	Positive (%)	Negative (%)	Positive (%)
HC2 test	46 (14.9)	263 (85.1)	23 (4.6)	476 (95.4)	4 (5.8)	65 (94.2)
Genotyping	97 (24.0)	307 (76.0)	19 (4.7)	388 (95.3)	12 (7.9)	139 (92.1)
Total	143 (20.1)	570 (79.9)	42 (4.6)	864 (95.4)	16 (7.3)	204 (92.7)

*HR-HPV* high-risk human papillomavirus, *CIN* cervical intraepithelial neoplasia, *ICC* invasive cervical cancer, *HC2* Hybrid Capture 2

### Prior co-testing results

Among the patients who underwent both cytology and HR-HPV co-testing, the histological diagnoses were as follows: CIN1, 556 patients; CIN2/3, 524 patients; and ICC, 230 patients. HC2 testing was performed in 194 (34.9%) of the 556 CIN1 patients, 213 (40.6%) of the 524 CIN2/3 patients, and 41 (17.8%) of the 230 ICC patients; the remaining patients underwent HPV genotyping (Table 4). The average interval between histological diagnosis and co-testing was 15.8 days (range, 0–90 days), 17.3 days (range, 0–127 days), and 10.4 days (range, 0–80 days) in the CIN1, CIN2/3, and ICC groups, respectively. The rate of abnormal cytological results was significantly lower in the CIN1 group (87.9%, 489/556) than in the CIN2/3 (92.7%, 486/524; *P* = 0.008) and ICC groups (93.5%, 215/230; *P* = 0.021). The overall HR-HPV prevalence in the CIN2/3 (92.7%) and ICC (94.8%) groups was similar to each other (*P* = 0.718) but significantly higher than that in the CIN1 group (72.8%, all *P* < 0.001). HC2 testing and genotyping detection had similar rates of HR-HPV detection in the CIN1 (76.3% vs. 71.0%, respectively; *P* = 0.181), CIN2/3 (93.9% vs. 92.0%, *P* = 0.401), and ICC (97.6% vs. 94.2%, *P* = 0.377) groups. The rate of abnormal findings on co-testing was significantly higher in the CIN2/3 (99.8%, *P* < 0.001) and ICC groups (99.6%, *P* = 0.003) than in the CIN1 group (95.3%), but the rates were similar in the former two groups (*P* = 0.549).

**Table 4:** Results of cytology and HR-HPV co-testing

Cytological interpretation	HR-HPV testing				Total	
	HC2 testing		Genotyping			
	Negative (%)	Positive (%)	Negative (%)	Positive (%)	Negative (%)	Positive (%)
CIN1 (*n* = 556)						
NILM	3 (1.5)	14 (7.2)	23 (6.4)	27 (7.5)	26 (4.7)	41 (7.4)
ASC-US	20 (10.3)	49 (25.3)	40 (11.0)	91 (25.1)	60 (10.8)	140 (25.2)
ASC-H	2 (1.0)	4 (2.1)	3 (0.8)	3 (0.8)	5 (0.9)	7 (1.3)
LSIL	18 (9.3)	79 (40.7)	38 (10.5)	130 (35.9)	56 (10.1)	209 (37.6)
HSIL	3 (1.5)	2 (1.0)	1 (0.3)	6 (1.7)	4 (0.7)	8 (1.4)
Total	46 (23.7)	148 (76.3)	105 (29.0)	257 (71.0)	151 (27.2)	405 (72.8)
CIN2/3 (*n* = 524)						
NILM	0 (0)	12 (5.6)	1 (0.3)	25 (8.0)	1 (0.2)	37 (7.1)
ASC-US	2 (0.9)	33 (15.5)	7 (2.3)	53 (17.0)	9 (1.7)	86 (16.4)
ASC-H	2 (0.9)	35 (16.4)	2 (0.6)	22 (7.1)	4 (0.8)	57 (10.9)
LSIL	4 (1.9)	52 (24.4)	6 (1.9)	74 (23.8)	10 (1.9)	126 (24.0)
HSIL	5 (2.3)	68 (31.9)	9 (2.9)	112 (36.0)	14 (2.7)	180 (34.4)
Total	13 (6.1)	200 (93.9)	25 (8.0)	286 (92.0)	38 (7.3)	486 (92.7)
ICC (*n* = 230)						
NILM	0 (0)	3 (7.3)	1 (0.5)	6 (3.2)	1 (0.4)	9 (3.9)
Unsatisfactory	0 (0)	0 (0)	0 (0)	5 (2.6)	0 (0)	5 (2.2)
ASC-US	1 (2.4)	3 (7.3)	3 (1.6)	15 (7.9)	4 (1.7)	18 (7.8)
ASC-H	0 (0)	10 (24.4)	1 (0.5)	29 (15.3)	1 (0.4)	39 (17.0)
LSIL	0 (0)	0 (0)	0 (0)	1 (0.5)	0 (0)	1 (0.4)
HSIL	0 (0)	18 (43.9)	1 (0.5)	100 (52.9)	1 (0.4)	118 (51.3)
AGC	0 (0)	0 (0)	2 (1.1)	3 (1.6)	2 (0.9)	3 (1.3)
Cancer cells	0 (0)	6 (14.6)	3 (1.6)	19 (10.1)	3 (1.3)	25 (10.9)
Total	1 (2.4)	40 (97.6)	11 (5.8)	178 (94.2)	12 (5.2)	218 (94.8)

*HR-HPV* high-risk human papillomavirus, *CIN* cervical intraepithelial neoplasia, *ICC* invasive cervical cancer, *NILM* negative for intraepithelial lesion or malignancy, *ASC-US* atypical squamous cells of undetermined significance, *ASC-H* atypical squamous cells-cannot exclude HSIL, *LSIL* low-grade squamous intraepithelial lesion, *HSIL* high-grade squamous intraepithelial lesion, *AGC* atypical glandular cells, *HC2* Hybrid Capture 2

### Comparison of different screening modalities

A comparison of the sensitivities of cytology alone, HR-HPV testing alone, and co-testing with both cytology and HR-HPV detection is shown in Table 5. In the CIN1 group, cytology alone (92.9, 95% confidence interval [CI]: 90.7–95.0) was significantly more sensitive than HR-HPV testing alone (79.9, 95% CI: 77.0–82.9; *P* < 0.001). In the same group, co-testing (95.3, 95% CI: 93.6–97.1) was significantly more sensitive than HR-HPV testing alone (*P* < 0.001) but only slightly more sensitive than cytology alone (*P* = 0.105). In the CIN2/3 group, the sensitivity of cytology alone (97.2, 95% CI: 96.1–98.3) was marginally higher than that of HR-HPV testing alone (95.4, 95% CI: 94.0.0–96.7; *P* = 0.062). However, the sensitivity of the combination (99.8, 95% CI: 99.4–100) was significantly higher than that of cytology alone (*P* = 0.001) and HR-HPV testing alone (*P* < 0.001). In the ICC group, HR-HPV testing alone (92.7, 95% CI: 89.3–96.2) was slightly, but not significantly more sensitive than cytology alone (89.1, 95% CI: 85.1–93.2, *P* = 0.185), while co-testing (99.6, 95% CI: 98.7–100) was significantly more sensitive than cytology alone (*P* < 0.001) and HR-HPV testing alone (*P* < 0.001). Furthermore, when 6 cases of unsatisfactory cytological interpretation were excluded (these cases needed to be resampled according to the guidelines [12, 15]), cytology alone (91.5% [205/224], 95% CI: 87.9–95.2) had very similar screening efficiency to HR-HPV testing alone (*P* = 0.636).

**Table 5:** Comparison of cytology alone, HR-HPV testing alone, and co-testing

Screening modality	CIN1 (***n*** = 1829)		CIN2/3 (***n*** = 2308)		ICC (***n*** = 680)	
	Sensitivity, %	95% CI	Sensitivity, %	95% CI	Sensitivity, %	95% CI
Cytology alone	92.9 (520/560)	90.7–95.0	97.2 (854/879)	96.1–98.3	89.1 (205/230)	85.1–93.2
HR-HPV testing alone	79.9 (570/713)	77.0–82.9	95.4 (864/906)	94.0–96.7	92.7 (204/220)	89.3–96.2
Co-testing	95.3 (530/556)	93.6–97.1	99.8 (523/524)	99.4–100	99.6 (229/230)	98.7–100

*HR-HPV* high-risk human papillomavirus, *CIN* cervical intraepithelial neoplasia, *ICC* invasive cervical cancer, *CI* confidence interval

## Discussion

The present study demonstrated that cervical cytology alone and HR-HPV testing alone had similar efficiency for CIN2/3 and ICC detection, while cervical cytology alone was significantly more sensitive than HR-HPV testing alone for CIN1 detection. Additionally, the study showed that compared to either method alone, combined co-testing could further slightly increase the screening efficiency for CIN1, CIN2/3, and ICC. All of the above data were obtained after the implementation of a systematic training and QC program for cervical cytology screening in Shandong Province, China, and the population of women tested had not previously undergone intensive screening. These results suggest that a systematic cervical cytology training and QC program can improve the screening efficiency of cervical cytology for the detection of CIN1, CIN2/3, and ICC, so that it is equivalent to or even slightly higher than that of HR-HPV detection.

A biopsy diagnosis of a CIN2/3 or worse lesion is the clinical threshold leading to ablative or excisional therapy. The treatment of CIN1 lesions, which have substantial rates of spontaneous regression, is discouraged, particularly in adolescents [15, 30]. However, it is imperative to closely follow CIN1 patients up, as the cumulative incidence of CIN2/3 or worse lesions is very high among CIN1 patients, especially among those with HR-HPV infection [30, 31]. Cytology and/or HR-HPV testing is recommended for follow-up evaluations [15, 30]. Thus far, limited data are available on the screening effectiveness of cytology and/or HR-HPV testing for the detection of CIN1. In our study, the sensitivity of cytology alone (92.9%) was significantly higher than that of HR-HPV testing alone (79.9%) in the CIN1 group. Furthermore, adding HR-HPV testing to cytology (95.3%) provided only a small and statistically insignificant increase in the screening sensitivity for CIN1 detection. In a large-scale summary of meta-analyses, Arbyn et al. [32] reported that a reflex HC2 test does not have a significantly higher sensitivity and has a significantly lower specificity than a repeat Pap smear for the triaging of women with LSIL. About 20–30% of patients with CIN1 test positive for only low-risk HPV [32, 33], and this may explain why HR-HPV testing alone had a low screening efficacy and why adding HR-HPV testing to cytology did not significantly improve the screening effectiveness for CIN1 detection. However, clinical studies have found that women with LSIL accompanied with low-risk HPV infection or no HPV infection rarely progress to CIN2/3 or worse lesions [31, 34]. Given that CIN1 progression is closely related to HR-HPV infection, HR-HPV testing, like cervical cytology, has high clinical utility in CIN1 screening.

Our data revealed that the rate of abnormal results was marginally higher for cytology alone (97.2%) than for HR-HPV testing alone (95.4%) in the CIN2/3 group, which is comparable to the data from the Cytopathology Department of Guangzhou KD (GKD), which is the headquarters of KD with full CAP certification in China [35]. GKD is in strict conformity with the laboratory workload standards and QC practices issued in the CAP Laboratory Accreditation Program checklists [10]. According to the GKD data, 93.1% patients had abnormal cytological results, and 91.7% patients had positive HR-HPV testing results within 6 months prior to the histological diagnosis of CIN2/3. Another study from West China also reported that the rate of positive results was significantly higher for cytology (95.7%) than for HR-HPV testing (89.9%) in 1094 CIN2/3 patients [36]. The screening results of CIN2/3 patients in this study and the two other studies [35, 36] from China are inconsistent with the results from dozens of randomized, controlled trials, which found that HR-HPV testing is more sensitive than cytology for identifying cervical cancer and its precursors during population screening [14, 37–39]. Additionally, a study of 14,261 cases from multiple US clinical centers [40] reported a 91.4% rate of positive results for cervical cytology and a 95.8% rate for HR-HPV testing performed within 1 year prior to the histological diagnosis; these results are discordant with the above Chinese data. This difference might be attributed to the fact that the populations from China had very limited prior screening, whereas the populations of developed countries largely undergo routine periodic screening. For those women who were never or rarely screened, CIN2/3 lesions might be diagnosed at much larger sizes than the lesions detected in women who underwent regular screening [41]. Therefore, more exfoliated neoplastic cells can be collected for making a definite interpretation, and the screening effectiveness of cytology might be higher in the underserved women than in the routinely screened women.

The present screening data showed that HR-HPV testing alone (92.7%) was only slightly more sensitive than cytology alone (89.1%) and without statistical significance in ICC patients, especially when cases of unsatisfactory interpretation had been excluded (91.5%). One retrospective study of GKD data [42] examined the screening results of 155 Chinese women who were diagnosed with ICC within 1 year after undergoing cervical cancer screening. The results of the GKD study showed that cervical cytology was significantly more sensitive than HR-HPV testing, with the rate of negative results being 1.9% for cytology and 9.7% for HR-HPV testing [42]. In contrast, the rate of negative cytological findings was as high as 15.5% among patients subsequently diagnosed with ICC in both the first (238 patients) [43] and second (161 patients) [36] largest women’s hospitals in China, in which no systematic training and QC processes were implemented. The rates of negative HR-HPV testing resulting in these two hospitals were 15.5 and 12.4%, respectively, which are much higher than the 7.3% rate in the present study and the 9.7% rate reported in the GKD study [42]. This difference in the screening efficacy of cytology and HR-HPV testing for ICC detection might be attributed to the high-quality cytology services offered after the establishment of systemic training and QC programs at JKD and GKD. Interestingly, a high rate of negative cytological results of 13.7%, and a concurrent 10.8% rate of negative HR-HPV testing were reported in a large US study involving the co-testing of 600 patients who were eventually diagnosed with ICC [44]. As mentioned above, one possible explanation might be due to differences in populations that did or did not undergo regular screening. For the largely underserved Chinese women, ICC lesions might be diagnosed at much larger sizes and/or at later stages [41].

Co-testing has been shown to not only detect significantly more CIN2/3 or worse lesions but also results in significantly lower rates of ICC and its precursor lesions in subsequent rounds of screening [13, 14, 17]. In the present study, we also found that the cytology and HR-HPV combination (99.8%) had significantly higher sensitivity than either cytology alone or HR-HPV testing alone in CIN2/3 detection, which is consistent with the three aforementioned retrospective studies from China [35, 36] and the US [40], which reported rates of positive co-testing results in 98.1, 99.6, and 99.4% of cases, respectively. Furthermore, co-testing (99.6%) in this study was significantly more sensitive than cytology alone and HR-HPV testing alone for ICC detection. These results are in accordance with retrospective studies from other daily clinical practices [36, 40, 42–44] and the aforementioned randomized controlled trials [13, 14, 17].

As a cervical cancer screening method, cytology has several advantages, such as simple preparation, low infrastructure requirements, and cost-effectiveness. In addition, the detection of different cytological abnormalities is helpful for the clinical triage of patients. Even now, cervical cytology (both LBC and CPS) is widely used for the large-scale screening and follow-up of high-risk groups, especially, in areas with poor economic conditions in China [9, 10, 25]. Our present data and those of several other Chinese studies [35, 36, 42, 43, 45] have demonstrated that cytology has similar or even slightly higher efficiency than HR-HPV testing for CIN2/3 and ICC screening. However, the interpretation of cervical cytological results involves a certain degree of subjectivity and requires specially trained and qualified cytotechnologists and/or cytopathologists. Moreover, strict QC standards are required to ensure the accuracy of interpretation. Developed countries have established well-organized cervical cytology training and QC programs to guarantee the efficiency of cytological screening [4, 5]. The data of this study and the GKD data [35, 42] indicate that systematic training and QC programs can markedly increase the screening effectiveness of cervical cytology in China. Of note, many challenges still exist in using cytology as a first-line method for cervical cancer screening in China. Even in Shandong Province, which has a relatively developed economy, systematic training programs for cytopathologists and standardized QC systems have not yet been widely established, and cervical cancer screening is not routinely carried out. A recent population-based, prospective study [46] from China reported a screening sensitivity of less than 71.1% for cervical cytology in the detection of CIN2/3 and ICC. These factors are the main reason for the high incidence and mortality of cervical cancer in China [23, 24].

In addition to accelerating the training of cytopathologists and establishing a cytological QC system to improve the efficiency of cervical cytology, it is necessary to seek alternative screening methods and gradually reduce the dependence on qualified cytopathologists. Compared with cytological screening, HR-HPV detection is easy to automate and establish QC processes for, which would ensure the accuracy of screening. Therefore, HR-HPV testing as a first-line screening method for cervical cancer has high application value in areas where there is a lack of qualified cytopathologists in China. However, we should also note that HR-HPV detection alone as a first-line screening method for cervical cancer has some limitations. Usually, most of the HPV load will automatically be eliminated by the host immune system within 8–10 months [2, 3]. Only a few persistent infections have the possibility of developing into cervical precancerous lesions and cervical cancer [2, 3]. This results in an inherent drawback in using HR-HPV detection as a first-line screening method [47]. Clinical studies [13, 46] have confirmed that the specificity and positive predictive value of HR-HPV testing are lower than those of cytological screening. According to the requirements of the American Society for Colposcopy and Cervical Pathology, women with positive HR-HPV test results need further colposcopy or cytology [15]. A recent retrospective analysis of 94,489 HPV genotyping results [29] showed that the rate of positive HR-HPV testing results was 24.2% in women from Shandong Province. Such a large number of HR-HPV-positive women, in most of whom the viral infection may be naturally eliminated by their own immune system, complicates the formulation of further treatment plans and raises concerns about excessive colposcopy and treatments for self-limiting HPV infections. Therefore, it is necessary to find new markers to separate out the cases of CIN2/3 or cervical cancer in HR-HPV-positive women and improve the efficiency of HR-HPV screening. In addition, HPV testing costs were about three times as much as cytology, and would place a heavy economic burden on underdeveloped regions, especially in suburban and rural areas. Moreover, a dozen HPV detection kits are currently used in clinical practice in China. The clinical validity and utility of these kits have not been fully verified using large-scale clinical trials, leading to a wide spectrum of difference in the first-line screening performance of these kits for cervical cancer.

## Conclusions

In conclusion, the results of this study show that the screening effectiveness of cervical cytology after the implementation of a systematic training and QC program was similar or even slightly higher than that of HR-HPV testing for the detection of CIN1, CIN2/3, and ICC in a largely unscreened population from Shandong Province, China. The experience of JKD can provide a good example to create training programs for cytopathologists and QC standards for cervical cancer screening in this region. In addition, new screening methods, such as HR-HPV detection, can be adopted as a favored alternative to cervical cytology, which may gradually reduce the dependence on cytological screening and rapidly popularize cervical cancer screening among women in low-resource settings such as rural areas from Shandong Province, China.

## Data Availability

All relevant data are within the paper. The data underlying this study are available and researchers may submit data requests to the the corresponding author on reasonable request.

## Abbreviations

JKD: Jinan KingMed Diagnostics
QC: Quality control
CIN: Cervical intraepithelial neoplasia
ICC: Invasive cervical cancer
CPS: Conventional Papanicolaou smear
LBC: Liquid-based cytology
HR-HPV: high-risk human papillomavirus
KD: KingMed Diagnostics
CAP: College of American Pathologists
HC2 assay: Hybrid Capture 2 assay
NILM: Negative for intraepithelial lesion or malignancy
ASC-US: Atypical squamous cells of undetermined significance
LSIL: Low-grade squamous intraepithelial lesion
HSIL: High-grade squamous intraepithelial lesion
ASC-H: Atypical squamous cells—cannot exclude HSIL
AGC: Atypical glandular cells
PCR: Polymerase chain reaction
GKD: Guangzhou KingMed Diagnostics
